# Bringing mindfulness to romantic relationships: linking attachment insecurity to young adults’ relationship satisfaction

**DOI:** 10.3389/fpsyg.2026.1732002

**Published:** 2026-08-28

**Authors:** Nureda Taşkesen, Funda Barutçu Yıldırım

**Affiliations:** 1 Department of Psychology, Norwegian University of Science and Technology, Trondheim, Norway; 2 Department of Educational Sciences, Middle East Technical University, Ankara, Türkiye

**Keywords:** anxious attachment, avoidant attachment, relationship well-being, trait mindfulness, relational mindfulness, mediation analysis, emerging adults, Turkey

## Abstract

**Introduction:**

Romantic relationships play a pivotal role in young adulthood, and attachment insecurity has consistently been linked to lower romantic relationship satisfaction. Mindfulness- and compassion-based resources may be associated with this link, helping to clarify how satisfaction varies among insecurely attached individuals. Building on this idea, the present study examined whether dispositional mindfulness, relationship mindfulness, and self-compassion are indirectly associated with the link between insecure attachment (anxiety and avoidance) and relationship satisfaction.

**Methods:**

We recruited 521 young adults in nonmarital romantic relationships in Türkiye (70% women; *M*_age_ = 22.52, *SD*_age_ = 2.45, range = 19–29). Participants completed the Relationship Assessment Scale, Experiences in Close Relationships–Revised, Mindful Attention Awareness Scale, Relationship Mindfulness Measure, and Self-Compassion Scale–Short Form.

**Results:**

Using structural equation modeling, we found that both attachment anxiety and avoidance were negatively associated with dispositional mindfulness (β_anxiety_ = −0.28, β_avoidance_ = −0.15), relationship mindfulness (β_anxiety_ = −0.38, β_avoidance_ = −0.13), and self-compassion (β_anxiety_ = −0.49, β_avoidance_ = −0.11). Higher relationship mindfulness, in turn, was linked to higher relationship satisfaction (β = 0.21). Notably, relationship mindfulness demonstrated a significant indirect association linking attachment anxiety to relationship satisfaction (β = −0.08). Dispositional mindfulness and self-compassion did not exhibit significant indirect associations.

**Discussion:**

These results point to relationship mindfulness as a relevant correlate of relationship satisfaction, with a small but significant indirect association observed specifically for individuals with higher attachment anxiety, underscoring the value of context-specific mindfulness in future research.

## 1 Introduction

Romantic relationships have long been recognized as a key context for understanding young adults’ psychological well-being. They are consistently linked to indicators of well-being and behavioral adjustment during the transition to adulthood (Arnett, 2000; Davila, 2010; Manning et al., 2010). Erikson (1977) identified establishing a healthy romantic relationship as one of the major developmental tasks of young adulthood. Longitudinal evidence supports this view: in a nine-year study following individuals from adolescence into young adulthood, romantic relationships became increasingly serious, committed, and more strongly tied to psychosocial adjustment compared to earlier years (Collibee and Furman, 2015). The same study further showed that, during young adulthood, higher-quality relationships served as protective factors against internalizing and externalizing symptoms and substance use.

Romantic partners also increasingly function as a secure base, a role long emphasized in attachment theory (Hazan and Shaver, 1987). Attachment theory (Bowlby, 1969) highlights the foundational role of early caregiver–child bonds in shaping later romantic relationship quality. According to the theory, these early interactions give rise to internal working models, which are cognitive and emotional representations of relationships, that are either secure or insecure (Bretherton, 1985). When caregivers are consistently available, warm, and responsive, children tend to form secure working models, learning that others can be trusted. In contrast, cold, inconsistent, or threatening caregiving can foster perceptions of others as unreliable sources of comfort and safety, leading to the development of insecure attachment strategies to manage psychological distress (Bowlby, 1969).

In adulthood, insecure internal working models typically manifest as anxious or avoidant attachment styles (Mikulincer and Shaver, 2003). Anxiously attached individuals often rely on hyperactivating strategies, characterized by an intense need for closeness, dependence on partners, heightened sexual attraction, jealousy, and fear of abandonment, whereas avoidantly attached individuals tend to use deactivating strategies, marked by fear of intimacy and commitment, reluctance to depend on others, an inflated sense of self, and difficulty accepting their partners’ shortcomings (Berant et al., 2005; Hazan and Shaver, 1987; Mikulincer and Shaver, 2003).

Both anxious and avoidant attachment are linked to lower relationship satisfaction in adulthood and have been found to remain relatively stable across the lifespan (Fraley et al., 2011; Li and Chan, 2012). While generally enduring, attachment patterns can shift under certain conditions. For instance, a pilot study in Spain found that attachment-based compassion therapy promoted changes from insecure to secure attachment, mediated by increases in self-compassion (Navarro-Gil et al., 2020). Identifying such psychological resources may help insecurely attached individuals with low relationship satisfaction adjust more successfully to adulthood through healthier romantic relationships.

Because attachment patterns tend to be relatively stable and not easily altered directly (Fraley et al., 2011; Mikulincer and Shaver, 2003), it may be more productive to examine capacities that can be cultivated across the lifespan and that plausibly help individuals manage the distressing internal experiences associated with insecure attachment (Kabat-Zinn, 2005). Three such capacities; dispositional mindfulness, relationship mindfulness, and self-compassion, are of particular interest here. Each has been linked to both intrapersonal and interpersonal well-being, yet each also tends to be lower among individuals with insecure attachment (Kimmes et al., 2018; Raque-Bogdan et al., 2011; Stevenson et al., 2017), raising the possibility that these capacities help explain, at least in part, why insecure attachment is associated with lower relationship satisfaction. Below, we consider each construct and its expected role in turn.

Mindfulness, broadly defined as “paying attention in a particular way; on purpose, in the present moment, and nonjudgmentally” (Kabat-Zinn, 1994), reflects a general, cross-situational capacity for present-moment attention. It has been associated with numerous benefits such as improved emotion regulation (Grossman et al., 2004), greater support exchange between partners (Karremans et al., 2017; Wachs and Cordova, 2007), more constructive conflict resolution (Barnes et al., 2007), and higher romantic relationship satisfaction (McGill et al., 2020). Yet, individuals with insecure attachment may struggle to sustain these benefits, as defensive processing can limit open, receptive awareness in close relationships (Ryan et al., 2007; Stevenson et al., 2017). Specifically, the hyperactivating strategies typical of anxious attachment may divert attention from the present moment and foster judgmental interpretations of experience, thereby undermining openness and nonreactivity, whereas the deactivating strategies characteristic of avoidant attachment may reduce emotional engagement and dampen awareness of both positive and negative experiences, thereby limiting the receptive attention central to mindfulness (Brown and Ryan, 2003; Mikulincer and Shaver, 2003; Stevenson et al., 2017). Thus, the very strategies that insecurely attached individuals rely on to regulate distress may also limit their capacity for mindfulness in both intrapersonal and relational contexts.

Because dispositional mindfulness reflects general attentional tendencies, it may not fully capture interpersonal processes within close relationships. This highlights the importance of examining relationship mindfulness, a construct that captures the application of mindfulness within romantic contexts. Specifically, it refers to intentionally bringing present-moment, nonjudgmental awareness to interactions with a partner (Kimmes et al., 2018) and has been associated with greater relationship satisfaction (Stanton et al., 2021), more frequent positive relationship behaviors (Gazder and Stanton, 2020), higher sexual satisfaction (Fincham, 2022), and enhanced partner well-being (Kimmes et al., 2020). In related work, relationship mindfulness moderated the association between perceived stress and negative relationship quality (Zheng et al., 2025), such that this link was weaker for those with higher levels of relationship mindfulness. A two-wave longitudinal dyadic study further demonstrated its incremental validity: relationship mindfulness, but not dispositional mindfulness, predicted increases in perceived partner responsiveness over 2 weeks, which in turn led to improvements in relationship quality over 2 months (Stanton et al., 2021). Moreover, insecure attachment has been linked to lower levels of relationship mindfulness (Gazder and Stanton, 2020). This pattern parallels evidence on dispositional mindfulness, further suggesting that attachment insecurity is associated with reduced capacity for mindfulness across both intrapersonal and relational contexts.

In addition to mindfulness, a related but conceptually distinct construct is self-compassion, which involves treating oneself with kindness, acceptance, and understanding during moments of difficulty or perceived inadequacy (Neff and Beretvas, 2013; Neff and Tirch, 2013). Self-compassion and mindfulness share an emphasis on awareness and nonjudgmental acceptance of inner experiences, which together support emotional well-being (Marshall and Brockman, 2016). Consistent with this, higher levels of self-compassion have been linked to lower levels of anxiety and depression (Joeng et al., 2017), more effective distress coping mechanisms (Bluth and Blanton, 2014; Mackintosh et al., 2018), and greater romantic relationship functioning (Barutçu Yıldırım et al., 2021; Baker and McNulty, 2011; Lathren et al., 2021).

Within intimate relationships, self-compassionate individuals were described by their partners as warmer and more considerate and tended to feel more emotionally connected, accepting, and willing to compromise during conflicts, resulting in less turmoil and greater relational well-being (Neff and Beretvas, 2013; Yarnell and Neff, 2013). Longitudinal and dyadic findings also suggested that self-compassion buffers against declines in relationship satisfaction, with some gender differences in how these associations unfold (Baker and McNulty, 2011; Lathren et al., 2021). Despite these benefits, insecure attachment is consistently linked to lower self-compassion (Lathren et al., 2021; Raque-Bogdan et al., 2011), which may contribute to relational difficulties by reducing individuals’ capacity to respond to personal shortcomings or relationship challenges with understanding rather than self-criticism. Importantly, as noted earlier, intervention research suggested that cultivating self-compassion may facilitate positive attachment change in romantic relationships (Navarro-Gil et al., 2020). These findings suggest that self-compassion supports individual and relational well-being and may also be a relevant correlate in the association between insecure attachment to relationship satisfaction.

Overall, although attachment insecurity is consistently linked to lower relationship satisfaction, it remains important to examine whether this association may also be partly driven by lower levels of dispositional mindfulness, relationship mindfulness, and self-compassion. If so, these skills, unlike the relatively stable patterns of attachment, represent capacities that can be cultivated and strengthened, and may therefore offer a promising focus for understanding correlates of relational well-being.

Based on the reviewed literature, the present study adopts a mediational framework, consistent with attachment theory’s developmental account: insecure attachment is theorized to shape individuals’ capacity for present-moment awareness and self-directed compassion over time (Mikulincer and Shaver, 2003), such that reduced mindfulness and self-compassion may represent a downstream consequence of attachment insecurity that may help explain its association with relationship satisfaction. In line with this framework, we expected attachment anxiety and avoidance to be associated with lower levels of dispositional mindfulness (Hypothesis 1a–b), relationship mindfulness (Hypothesis 2a–b), and self-compassion (Hypothesis 3a–b), which would, in turn, relate to lower relationship satisfaction (Hypothesis 4a–c). Thus, we hypothesized a parallel multiple mediation model, in which the associations from attachment anxiety and avoidance to relationship satisfaction would be mediated by dispositional mindfulness (Hypothesis 5a–b), relationship mindfulness (Hypothesis 6a–b), and self-compassion (Hypothesis 7a–b). In addition, we included gender as a covariate in our main model where MANOVA (followed by individual ANOVAs) indicated significant group differences, as suggested by previous research (Kimmes et al., 2020; Lathren et al., 2021; Yarnell et al., 2015).

## 2 Materials and methods

### 2.1 Participants

This study targeted young adults aged 18–29 years residing in Türkiye who had been in a committed, nonmarital romantic relationship for at least 1 month. A total of 578 individuals accessed the study and provided informed consent. Following the application of our eligibility criteria, 20 participants were excluded for not meeting the age requirement, and 27 for not meeting the relationship status criteria. Further data screening identified nine univariate outliers and one multivariate outlier, which were subsequently removed. The final analytical sample consisted of 521 young adults (aged 19–29 years; *M* = 22.52, *SD* = 2.45), of whom 70.44% were women, 27.06% were men, and 2.5% were non-binary. All participants were university students (87.91% bachelor’s, 11.13% master’s, 0.96% PhD) and had been in a committed, nonmarital romantic relationship for at least 1 month, with relationship length ranging up to 96 months (*M* = 20.66, *SD* = 19.32).

### 2.2 Procedure

Data for this cross-sectional study were collected in Türkiye between May 2021 and January 2022 via an anonymous online survey platform, METUSurvey. Recruitment involved convenience and snowball sampling, using posters placed on the university campus and emails sent to students’ institutional addresses. Participation was voluntary, and no compensation was offered. Participants completed the questionnaires anonymously in Turkish after providing informed consent. The study protocol was approved by the Human Research Ethics Committee of Middle East Technical University (protocol nr. 133-2021, approved on 15 April 2021).

### 2.3 Measures

Internal consistency of the measures was assessed using both Cronbach (1951) alpha and McDonald (1999) omega. Reliability values ≥ 0.70 were considered acceptable, ≥ 0.80 good, and ≥ 0.90 excellent (Nunnally and Bernstein, 1994).

#### 2.3.1 Relationship assessment scale

This unidimensional seven-item scale (RAS; Hendrick, 1988; Curun, 2001) assesses romantic relationship satisfaction (e.g., “How well does your partner meet your needs?”). Items were rated on a 7-point Likert scale (1 = low satisfaction, 7 = high satisfaction), with two items reverse-coded so that higher scores indicate higher levels of satisfaction. The scale demonstrated good internal consistency in the present study (α = 0.86, ω = 0.87).

#### 2.3.2 Experiences in close relationships – revised

This 36-item scale (ECR-R; Fraley et al., 2000; Selçuk et al., 2005) measures two dimensions of insecure attachment in adults: attachment anxiety (18 items; e.g., “I often worry that my partner will not want to stay with me”) and attachment avoidance (18 items; e.g., “I do not feel comfortable opening up to romantic partners”). Items were rated on a 7-point Likert scale (1 = strongly disagree, 7 = strongly agree), with 14 items reverse-coded so that higher scores indicate higher levels of insecure attachment. Both subscales demonstrated good internal consistency in the present study (anxiety: α and ω = 0.88; avoidance: α = 0.86, ω = 0.87).

#### 2.3.3 Mindful attention awareness scale

The original 15-item unidimensional scale of dispositional mindfulness (MAAS; Brown and Ryan, 2003; Özyeşil et al., 2011) was used in a shortened 5-item form, developed through an Item Response Theory Analysis by Van Dam et al. (2010); e.g., “It seems I am running on automatic, without much awareness of what I’m doing”. Items were rated on a 6-point Likert scale (1 = almost never, 6 = almost always) and were reverse-coded so that higher scores indicate greater dispositional mindfulness. The scale demonstrated good internal consistency in the present study (α = 0.79, ω = 0.80).

#### 2.3.4 Relationship mindfulness measure

This five-item unidimensional scale (RMM; Kimmes et al., 2018; Taşkesen and Barutçu Yıldırım, 2024) assesses mindfulness in the context of romantic relationships (e.g., “I have conversations with my partner without being really attentive.”). Items were rated on a 6-point Likert scale (1 = almost never, 6 = almost always) and were reverse-coded so that higher scores indicate greater relationship mindfulness. The scale demonstrated good internal consistency in the present study (α and ω = 0.76).

#### 2.3.5 Self-compassion scale – short form

This unidimensional 12-item scale (SCS-SF; Raes et al., 2011; Barutçu Yıldırım et al., 2023) measures individuals’ compassion toward themselves (e.g., “When I’m going through a very hard time, I give myself the caring and tenderness I need.”). Raes et al. (2011) reported that the SCS-SF supports a single higher-order self-compassion factor in addition to its six subscales, and that total scores correlate near-perfectly with the original long-form SCS (*r* ≥ 0.97). Consistent with this, self-compassion was modelled as a single latent factor in the present study. Items were rated on a 5-point Likert scale (1 = almost never, 5 = almost always), with six items reverse-coded so that higher scores indicate greater self-compassion. The scale demonstrated good internal consistency in the present study (α and ω = 0.88).

### 2.4 Analysis plan

Prior to the main analyses, the data were screened for inclusion criteria, missing values, and outliers. Missing data were minimal: four cases (0.8%) each had one missing value across different RAS items, despite the forced-response survey format, likely due to a technical issue or the survey page closing during completion. Data quality was further supported by an embedded attention check item, which all participants answered correctly. Univariate outliers were identified using standardized z-scores, with a cutoff of ±3.29, and multivariate outliers were detected using Mahalanobis distances with a critical chi-square value at *p* < 0.001 (Tabachnick and Fidell, 2013). The assumptions of normality, multicollinearity, linearity, and homoscedasticity (Kline, 2016) were examined, and no violations were detected. Subsequently, descriptive statistics, internal consistency estimates, and bivariate correlations among study variables were computed.

Age, relationship length, and gender were examined as potential covariates of endogenous variables (mindfulness constructs, self-compassion, and relationship satisfaction), using bivariate correlations for age and relationship length, and MANOVA followed by individual ANOVAs for gender (see Sections 3.1.1 and 3.1.2 for these results and the covariate decisions). Because the non-binary subsample was small (*n* = 13, 2.5%) and insufficient for stable group comparisons, gender was coded as binary (women, men), and nonbinary participants were excluded from any analysis that included gender as a variable.

These decisions affected the sample size available for each analysis. The four cases with a missing RAS item were excluded from analyses requiring complete RAS item-level data (such as the measurement model and the item-level sensitivity model reported in Supplementary Table 1; *N* = 517) but were retained elsewhere using composite scores computed from available items. The structural model additionally excluded the 13 non-binary participants, for whom gender could not be coded on the binary covariate, yielding *N* = 504 for this analysis. Sample sizes for each reported analysis are noted in the corresponding table and figure.

For constructs with a relatively large number of items (i.e., 12 and 18 items), parceling was conducted to reduce model complexity and improve estimation, using the factorial algorithm method, which assigns items to parcels based on their factor loadings to ensure even distribution (Matsunaga, 2008). As a sensitivity check, we also estimated an item-level measurement model without parcels. Then, the measurement model was tested using confirmatory factor analysis (CFA), including all latent variables simultaneously. The hypothesized model was subsequently tested using structural equation modelling (SEM).

Although no *a priori* power analysis was conducted, the sample size available for the primary structural model (*N* = 504) exceeds recommended minimums for SEM (*N* ≥ 200; Kline, 2016) and is larger than the average sample size used in comparable studies on attachment and mindfulness (*N*_mean_ = 335; Stevenson et al., 2017). In addition, a simulation study on complex mediation SEMs (Sim et al., 2022) indicates that, for similar models, medium-sized effects typically require between 200 and 430 participants, suggesting that our sample size was adequate.

Correlation strengths were interpreted based on Cohen’s (1988) criteria, with values of r = 0.10, 0.30, and 0.50 indicating small, medium, and large effects, respectively. All CFA-based analyses were conducted using robust maximum likelihood estimation (MLR). Confidence intervals for all parameter estimates, including specific and total indirect effects, were computed using the delta method based on the robust (sandwich-corrected) standard errors produced under MLR estimation. Model fit was evaluated using multiple indices, including the *χ^2^* test, CFI, TLI, SRMR, and RMSEA. Acceptable-to-good model fit was determined by *χ^2^*/*df* ≤ 3, CFI and TLI ≥ 0.90, SRMR ≤ 0.10, and RMSEA ≤ 0.08 (Browne and Cudeck, 1992; Hu and Bentler, 1999; Kline, 2016). All reported model fit indices are robust estimates, and parameter estimates are standardized. The significance threshold for all statistical tests was set at *p* < 0.05. All analyses were conducted in RStudio (Posit Team, 2023). The analysis scripts are openly available at: https://osf.io/mc5v2/overview?view_only=c8066c95715649caa9384353a5e07e3e.

## 3 Results

### 3.1 Preliminary analyses

#### 3.1.1 Bivariate correlations

As shown in Table 1, all correlations among the main study variables were significant, ranging from small to large in magnitude. Attachment anxiety and avoidance were positively and moderately correlated, indicating that each predictor has unique variance in contributing to the model. Both dimensions of insecure attachment were negatively associated with relationship satisfaction, dispositional mindfulness, relationship mindfulness, and self-compassion, with the association between avoidance and relationship satisfaction being particularly strong. Relationship satisfaction was positively associated with both mindfulness variables and with self-compassion. Finally, the two mindfulness constructs were moderately correlated with one another, and both were positively associated with self-compassion.

**Table 1:** Descriptive statistics and correlations among the main study variables.

Variables	*M*	*SD*	1	2	3	4	5
1. Attachment anxiety	3.43	1.01					
2. Attachment avoidance	2.26	0.79	0.38				
3. Relationship satisfaction	6.10	0.82	−0.37	−0.53			
4. Dispositional mindfulness	4.32	0.99	−0.29	−0.24	0.19		
5. Relationship mindfulness	4.59	1.00	−0.36	−0.23	0.33	0.46	
6. Self-compassion	2.85	0.82	−0.48	−0.28	0.21	0.32	0.23

*N* = 521. All correlations were significant at *p* < 0.001.

As participants got older, their levels of relationship mindfulness and self-compassion increased (*r*s = 0.09, *p*s < 0.05), while their attachment anxiety decreased (*r* = −0.16, *p* < 0.001). Older participants also reported being in longer romantic relationships (*r* = 0.32, *p* < 0.001). Longer relationships were associated with lower attachment anxiety (*r* = −0.17, *p* < 0.001) and avoidance (*r* = −0.15, *p* < 0.001). Age and relationship length were not significantly associated with any other variables. Given that relationship length was not significantly associated with any endogenous variable, it was not retained as a covariate. Age showed statistically significant but negligible correlations with self-compassion and relationship mindfulness (*r*s = 0.09), falling below the threshold for even a small effect under the criteria adopted in this study (*r* = 0.10; Cohen, 1988), and was not significantly associated with relationship satisfaction; age was therefore also not retained as a covariate.

#### 3.1.2 Group differences by gender

A MANOVA was conducted to examine the gender differences associated with dispositional mindfulness, relationship mindfulness, self-compassion, and relationship satisfaction. The multivariate test was significant, indicating that the set of outcomes differed by gender (*N* = 508; *V* = 0.06, *F*_(4, 503)_ = 7.81, *p* < 0.001). Follow-up univariate ANOVAs (*N*s = 508) revealed a significant gender difference only for self-compassion (*F*_(1, 506)_ = 22.06, *p* < 0.001), with men (*M* = 3.14, *SD* = 0.70) reporting higher levels than women (*M* = 2.76, *SD* = 0.84). No significant gender differences were found for dispositional mindfulness (*F*_(1, 506)_ = 0.09, *p* > 0.05), relationship mindfulness (*F*_(1, 506)_ = 2.63, *p* > 0.05), or relationship satisfaction (*F*_(1, 506)_ = 0.26, *p* > 0.05). Based on these findings, gender was included as a covariate for self-compassion in the structural model.

### 3.2 Primary analyses

#### 3.2.1 Measurement model

For constructs with a relatively large number of items, the parceling was applied using the factorial algorithm method (see Table 2 for factor loadings). Attachment anxiety and avoidance were each represented by three parcels (six items per parcel), and self-compassion by four parcels (three items each). Mindfulness constructs were modeled with five individual items each, while relationship satisfaction was represented by seven items. The full measurement model, including all latent constructs, demonstrated good fit [*χ^2^* (309) = 856.36, *p* < 0.001, *χ^2^*/*df* = 2.77, SRMR = 0.07, RMSEA = 0.06; CFI = 0.91; TLI = 0.90]. Factor loadings ranged from 0.44 to 0.90, and all latent variables were significantly correlated (*p* < 0.001).

**Table 2:** Factor loadings for the measurement model.

Latent variable and indicators	*B*	*SE*	λ
Attachment anxiety			
Parcel 1	1.00	—	0.86
Parcel 2	0.89	0.04	0.84
Parcel 3	0.89	0.05	0.80
Attachment avoidance			
Parcel 1	1.00	—	0.71
Parcel 2	1.05	0.07	0.87
Parcel 3	1.24	0.08	0.90
Dispositional mindfulness			
Item 1	1.00	—	0.79
Item 2	0.90	0.06	0.73
Item 3	0.57	0.08	0.45
Item 4	0.95	0.05	0.81
Item 5	0.77	0.07	0.55
Relationship mindfulness			
Item 1	1.00	—	0.66
Item 2	0.80	0.07	0.62
Item 3	0.68	0.10	0.49
Item 4	0.92	0.08	0.71
Item 5	0.87	0.07	0.67
Self-compassion			
Parcel 1	1.00	—	0.78
Parcel 2	1.08	0.05	0.87
Parcel 3	1.04	0.05	0.82
Parcel 4	1.05	0.07	0.73
Relationship satisfaction			
Item 1	1.00	—	0.79
Item 2	1.15	0.06	0.90
Item 3	1.00	0.07	0.77
Item 4	0.65	0.08	0.55
Item 5	1.16	0.06	0.82
Item 6	0.37	0.06	0.44
Item 7	0.95	0.09	0.61

*N* = 517. All indicators were significant at *p* < 0.001. B, unstandardized factor loading; SE, standard error; λ, standardized factor loading.

As a sensitivity check, we also estimated an item-level measurement model without parcels. This model showed poorer fit than the parceled measurement model, with modification indices pointing primarily to sub-facet structure within the SCS and item-wording effects within the ECR-R (see Supplementary Table 1). Given these item-level sources of misfit and the theoretical focus of the present study on the higher-order constructs, we retained the parceled measurement model as primary.

#### 3.2.2 Structural model

The next step involved testing the hypothesized structural model, which included direct paths from attachment dimensions to relationship satisfaction and indirect paths through dispositional mindfulness, relationship mindfulness, and self-compassion. Gender was included as a covariate for self-compassion, given the significant group difference found in the preliminary analyses. The model demonstrated adequate fit on absolute fit indices, though CFI and TLI fell marginally below conventional thresholds [*χ^2^* (338) = 1016.47, *p* < 0.001, *χ^2^*/*df* = 3.01, SRMR = 0.08, RMSEA = 0.06, CFI = 0.89, TLI = 0.88].

**Figure 1:**
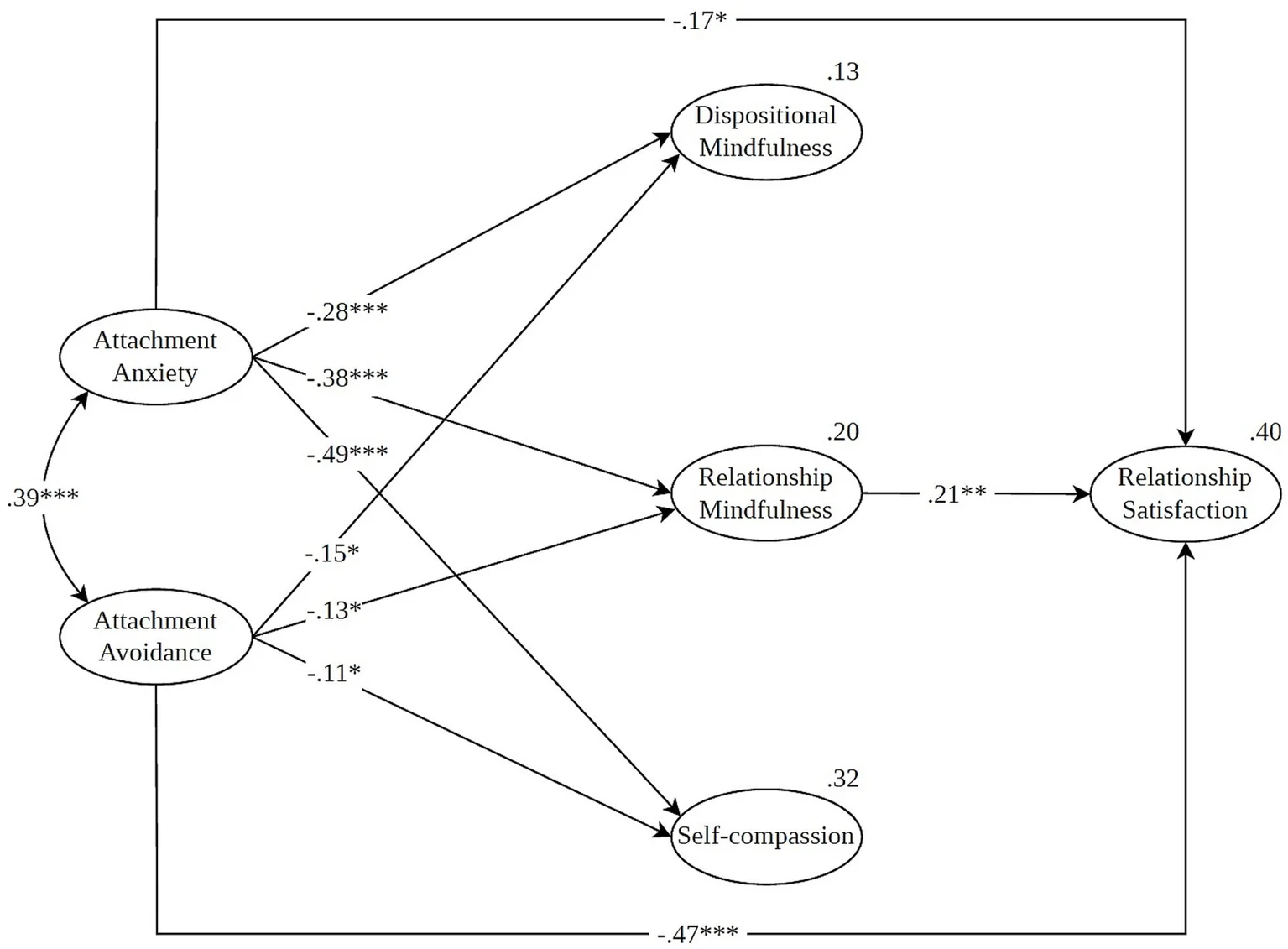
The structural model and the standardized estimates. *N* = 504. Standardized path coefficients (β) were significant at *p < 0.05, **p < 0.01, ***p < 0.001. *R^2^* values for each endogenous variable are shown in the upper right corner of the corresponding latent construct.

As shown in Figure 1, both dimensions of insecure attachment were significantly and negatively associated with all endogenous variables: dispositional mindfulness, relationship mindfulness, self-compassion, and relationship satisfaction. Gender significantly related to self-compassion (with men reporting higher levels on average). Relationship mindfulness was significantly and positively related to relationship satisfaction, but neither dispositional mindfulness nor self-compassion showed a significant association with satisfaction. Only one indirect path was significant. Attachment anxiety was associated with lower relationship satisfaction via lower relationship mindfulness (β = −0.08, *p* < 0.01, 95% CI [−0.119, −0.017]). The total effect of attachment anxiety on relationship satisfaction was significant and negative (β = −0.22, *p* < 0.001, 95% CI [−0.270, −0.094]), as was the total effect of attachment avoidance (β = −0.48, *p* < 0.001, 95% CI [−0.765, −0.455]). Overall, the structural model accounted for 40.1% of the variance in relationship satisfaction (see Table 3 for direct, indirect, total, and covariate effects in the structural model).

**Table 3:** Direct, indirect, total, and covariate effects in the structural model.

Direct, indirect, total, and covariate effects	*B*	*SE*	[95% CI]	β	*p*
Direct effects of attachment anxiety					
Attachment Anxiety → Dispositional Mindfulness	−0.29	0.07	[−0.43, −0.16]	−0.28	<0.001
Attachment Anxiety → Relationship Mindfulness	−0.39	0.07	[−0.53, −0.25]	−0.38	<0.001
Attachment Anxiety → Self-Compassion	−0.35	0.03	[−0.41, −0.28]	−0.49	<0.001
Attachment Anxiety → Relationship Satisfaction	−0.14	0.06	[−0.25, −0.04]	−0.17	<0.05
Direct effects of attachment avoidance					
Attachment Avoidance → Dispositional Mindfulness	−0.23	0.10	[−0.42, −0.03]	−0.15	<0.05
Attachment Avoidance → Relationship Mindfulness	−0.20	0.10	[−0.39, −0.00]	−0.13	<0.05
Attachment Avoidance → Self-Compassion	−0.12	0.05	[−0.22, −0.01]	−0.11	<0.05
Attachment Avoidance → Relationship Satisfaction	−0.59	0.08	[−0.75, −0.43]	−0.47	<0.001
Direct effects of mediators					
Dispositional Mindfulness → Relationship Satisfaction	−0.03	0.04	[−0.12, 0.05]	−0.04	>0.05
Relationship Mindfulness → Relationship Satisfaction	0.17	0.06	[0.07, 0.28]	0.21	<0.01
Self-Compassion → Relationship Satisfaction	−0.06	0.06	[−0.18, 0.06]	−0.05	>0.05
Indirect effects of attachment anxiety					
Attachment Anxiety → Dispositional Mindfulness → Relationship Satisfaction	0.01	0.01	[−0.02, 0.04]	0.01	>0.05
Attachment Anxiety → Relationship Mindfulness → Relationship Satisfaction	−0.07	0.03	[−0.12, −0.02]	−0.08	<0.01
Attachment Anxiety → Self-Compassion → Relationship Satisfaction	0.02	0.02	[−0.02, 0.06]	0.02	>0.05
Indirect effects of attachment avoidance					
Attachment Avoidance → Dispositional Mindfulness → Relationship Satisfaction	0.01	0.01	[−0.01, 0.03]	0.01	>0.05
Attachment Avoidance → Relationship Mindfulness → Relationship Satisfaction	−0.03	0.02	[−0.07, 0.01]	−0.03	>0.05
Attachment Avoidance → Self-Compassion → Relationship Satisfaction	0.01	0.01	[−0.01, 0.02]	0.01	>0.05
Total effects					
Attachment Anxiety → Relationship Satisfaction	−0.18	0.05	[−0.27, −0.09]	−0.22	<0.001
Attachment Avoidance → Relationship Satisfaction	−0.61	0.08	[−0.77, −0.46]	−0.48	<0.001
Covariate					
Gender → Self-Compassion	0.30	0.06	[0.19, 0.42]	0.19	<0.001

*N* = 504. B, unstandardized path coefficient; SE, standard error; CI, confidence interval; β, standardized path coefficient. The positive coefficient for gender indicates that men reported higher levels of self-compassion than women. CIs for all parameters were computed using the delta method, based on robust (sandwich-corrected) standard errors obtained under robust maximum likelihood (MLR) estimation.

## 4 Discussion

The present study examined whether dispositional mindfulness, relationship mindfulness, and self-compassion were associated with the well-established link between insecure attachment and relationship satisfaction in a sample of 521 young adults in Türkiye. SEM results indicated that both attachment anxiety and avoidance were associated with lower levels of dispositional mindfulness, relationship mindfulness, and self-compassion, consistent with expectations. In turn, relationship mindfulness was positively associated with relationship satisfaction, and a small but significant indirect association was observed between attachment anxiety and relationship satisfaction via relationship mindfulness. Contrary to expectations, neither dispositional mindfulness nor self-compassion was significantly associated with relationship satisfaction, and no indirect effects were observed for attachment avoidance. In the following sections, we address these partly expected and unexpected findings in more detail.

### 4.1 Linking attachment anxiety to mindfulness and self-compassion

In the present study, higher attachment anxiety was associated with lower levels of dispositional mindfulness, relationship mindfulness, and self-compassion, consistent with Hypotheses 1a, 2a, and 3a. These results converge with prior empirical work showing that attachment anxiety is consistently associated with lower mindfulness and self-compassion (Kimmes et al., 2018; Raque-Bogdan et al., 2011; Stevenson et al., 2017).

Theoretically, this pattern is consistent with the use of hyperactivating strategies, which involve heightened vigilance for rejection, rumination about relationship threats, and a tendency to exaggerate negative emotional cues from partners (Hazan and Shaver, 1987; Mikulincer and Shaver, 2003). Such preoccupation with relational threat may divert attentional resources from the present moment and foster defensive or judgmental interpretations of experience (Berant et al., 2005; Stevenson et al., 2017), thereby undermining openness and nonreactivity: skills central to both mindfulness and self-compassion (Brown and Ryan, 2003; Neff and Germer, 2013).

These intrapersonal difficulties with awareness and regulation may extend to relational contexts, consistent with the negative association of attachment anxiety with relationship mindfulness, which entails present-moment, nonjudgmental awareness of partner interactions (Kimmes et al., 2018). When attention is dominated by fears of abandonment and emotional reactivity (Berant et al., 2005), sustaining mindful engagement with a partner without judgment or defensiveness may become more difficult. In this sense, attachment anxiety may interfere with both general mindfulness capacities and their application in intimate relationships.

The negative link with self-compassion is also consistent with research showing that individuals high in attachment anxiety often hold a negative model of the self, perceive themselves as less worthy of love and support, and respond to personal shortcomings with self-criticism rather than self-kindness (Bartholomew and Horowitz, 1991; Berant et al., 2005; Raque-Bogdan et al., 2011). Taken together, our findings suggest that attachment anxiety may undermine mindful and compassionate awareness in both intrapersonal and relational domains, consistent with previous conceptual and empirical research (Kimmes et al., 2018; Raque-Bogdan et al., 2011; Stevenson et al., 2017).

### 4.2 Linking attachment avoidance to mindfulness and self-compassion

In the present study, greater attachment avoidance was associated with lower dispositional mindfulness, relationship mindfulness, and self-compassion, consistent with Hypotheses 1b, 2b, and 3b. Prior research has found similar patterns (e.g., Kimmes et al., 2018; Raque-Bogdan et al., 2011; Stevenson et al., 2017) and attachment theory provides a useful framework for understanding these associations.

Avoidantly attached individuals rely on deactivating strategies to regulate attachment needs, such as suppressing attachment-related thoughts and feelings, keeping emotional distance from partners, and prioritizing independence over closeness (Fraley and Shaver, 1997; Mikulincer and Shaver, 2003). These strategies may protect against perceived threats to autonomy but often result in reduced emotional engagement in relationships (Shaver and Mikulincer, 2002) and dampened intensity of both positive and negative emotional experiences (Li and Chan, 2012). As a result, the open, receptive awareness central to mindfulness (Brown and Ryan, 2003), whether general or relationship-specific may be undermined. Thus, the very strategies that shield avoidantly attached individuals from relational vulnerability (Mikulincer and Shaver, 2003) may simultaneously limit their capacity for mindfulness in both intrapersonal and interpersonal contexts.

These deactivating strategies may be relevant to understanding the negative link between attachment avoidance and self-compassion observed in our findings. By minimizing emotional exposure, deactivation can include suppressing recognition of one’s own vulnerabilities or shortcomings (Mikulincer and Shaver, 2003; Shaver and Mikulincer, 2002). This avoidance of self-relevant emotions might make it harder to respond to personal difficulties with kindness and nonjudgment, the hallmarks of self-compassion (Neff and Germer, 2013). Although attachment theory suggests that avoidant individuals tend to hold a relatively positive self-view and negative view of others (Bartholomew and Horowitz, 1991), this self-perception often reflects a defensive self-reliance rather than genuine self-acceptance (Raque-Bogdan et al., 2011; Shaver et al., 2016). When threatened by failure, this defensive stance may translate into self-criticism (Mikulincer and Shaver, 2003), consistent with the negative link between attachment avoidance and self-compassion found in our study as well as in prior work (Lathren et al., 2021).

Notably, our results also showed that attachment avoidance (β_range_ = −0.11 to −0.15) showed weaker associations with the mindfulness and self-compassion constructs than anxiety did (β_range_ = −0.28 to −0.49), replicating earlier findings. For example, Walsh et al. (2009) reported that attachment avoidance correlated with mindfulness but failed to predict overall mindfulness once attachment anxiety was accounted for. Similarly, mindfulness buffered the effects of attachment anxiety, but not avoidance, on relationship dissolution in longitudinal work (Saavedra et al., 2010). Pepping et al. (2014) further found that avoidance predicted fewer facets of mindfulness than anxiety, unaffected by meditation experience.

This weaker pattern for avoidance also extends to self-compassion, consistent with prior research showing stronger negative associations with attachment anxiety than avoidance (Bolt et al., 2019; Gerber et al., 2015; Raque-Bogdan et al., 2011; Wei et al., 2011). This distinction can be understood through Bowlby’s notion of internal working models of self, which reflect whether one sees oneself as worthy of love and support (Bartholomew and Horowitz, 1991). Anxiously attached individuals typically hold negative self-models, which are closely tied to self-critical tendencies and low self-compassion (Bolt et al., 2019; Hazan and Shaver, 1987; Wei et al., 2011). In contrast, avoidantly attached individuals often maintain relatively positive self-views that are defensive in nature, emphasizing independence, repressing insecurity, and attributing difficulties to others, rather than reflecting genuine self-acceptance (Berant et al., 2005; Hazan and Shaver, 1987; Mikulincer and Shaver, 2003; Shaver et al., 2016). Supporting this distinction, Bolt et al. (2019) found that attachment avoidance was more strongly linked to lower compassion for one’s partner than for oneself, whereas attachment anxiety was more clearly associated with lower self-compassion.

### 4.3 Links to relationship satisfaction

In the present study, relationship mindfulness was positively associated with relationship satisfaction, consistent with Hypothesis 4b, whereas dispositional mindfulness and self-compassion were not, contrary to Hypothesis 4a and 4c. Also, attachment anxiety was indirectly associated with lower satisfaction through lower relationship mindfulness, consistent with Hypothesis 6a. No other indirect associations were found, contrary to Hypotheses 5a–b (dispositional mindfulness), 6b (relationship mindfulness for avoidance), and 7a–b (self-compassion).

Because higher scores reflect greater attachment anxiety, greater relationship mindfulness, and greater relationship satisfaction, respectively, it is worth clarifying how these associations fit together. Individuals higher in attachment anxiety tended to report lower relationship mindfulness; in turn, lower relationship mindfulness was associated with lower relationship satisfaction. In other words, the indirect association suggests that attachment anxiety was linked to lower relationship satisfaction in part through its association with lower relationship mindfulness. We discuss this finding, along with the other direct and indirect associations examined in this study, below.

#### 4.3.1 Mindfulness

To start with results supporting our hypothesis, our findings showed that those who engage in relationship mindfulness tended to be more satisfied with their current romantic relationships. Consistent with this, lower relationship mindfulness characterized the indirect association between attachment anxiety and relationship satisfaction. To date, relatively few studies have investigated romantic relationship mindfulness; however, those that have consistently support these results, reflecting a rapidly growing body of literature.

For example, after Kimmes et al. (2018) introduced the relationship-specific mindfulness measure and found associations with greater relationship satisfaction, the literature has steadily grown, confirming and extending these findings by linking relationship mindfulness to higher positive and lower negative relationship quality, reduced stress, lower conflict intensity and frequency, and even improved physical health (Kimmes et al., 2020; Morris et al., 2024). Longitudinal work further demonstrated its unique predictive value: Stanton et al. (2021) found that relationship mindfulness, but not dispositional mindfulness, predicted increases in partner responsiveness and later improvements in relationship quality. Other recent studies highlighted its buffering role under stress and the importance of perceiving one’s partner as mindful, which can be as predictive, or even more predictive, of relationship outcomes than self-reports (Zheng et al., 2025; Kimmes et al., 2025). Other work using a different relationship-specific measure, the Mindfulness in Couple Relationships Scale, also found positive associations with higher relationship quality, positivity, and sexual satisfaction, as well as negative associations with relationship negativity, capturing links that dispositional mindfulness has not consistently shown (McGill et al., 2022). Together, these findings suggest that relationship mindfulness is a consistent correlate of relational well-being across multiple studies, with associations observed beyond those found for dispositional mindfulness.

On the other hand, our results did not support the link between dispositional mindfulness and relationship satisfaction, unlike the majority of published studies that report significant positive links (e.g., McGill et al., 2016; Quinn-Nilas, 2020). Several explanations may account for this unexpected pattern. First, as mentioned earlier, broad dispositional measures may be less predictive of relationship outcomes than context-specific ones. In particular, relationship mindfulness has been shown to capture unique relational processes: a two-wave longitudinal dyadic study found that relationship mindfulness, but not dispositional mindfulness, predicted increases in perceived partner responsiveness over 2 weeks, which in turn improved relationship quality over 2 months (Stanton et al., 2021). Similarly, other studies suggest that relationship-specific mindfulness measures more consistently account for variance in relational outcomes compared to dispositional scales (Kimmes et al., 2018; Leavitt et al., 2019; McGill et al., 2022).

Second, dispositional mindfulness may sometimes not directly translate into relational benefits unless accompanied by factors such as partner-focused acceptance, as shown by Kappen et al. (2018), who found indirect effects via partner acceptance but inconsistent or absent direct effects. Similarly, as noted by Quinn-Nilas (2020), meditation experience may be a critical moderator: meditators tend to score higher on dispositional mindfulness facets even after controlling for demographics (Baer et al., 2008), and the absence of such data in the present study may have masked group differences or introduced confounding.

In addition, culture might have played a role. As noted by Arpacıoglu et al. (2023), who reported unexpected negative association between dispositional mindfulness and relationship satisfaction in Turkish adults, higher dispositional mindfulness could increase the awareness of relational difficulties, thereby lowering relationship satisfaction. Relatedly, mindfulness has been linked to reduced prosociality among individuals with independent self-construals, and those primed with independence in a lab (Poulin et al., 2021), suggesting that social goals and cultural orientations might alter the associations of mindfulness. Türkiye, a Mediterranean society that blends Eastern and Western ideals and reflects a distinct combination of independent and interdependent orientations (Uskul et al., 2023), remains underrepresented in mindfulness research compared to Anglo-Western countries. This mirrors the broader dominance of WEIRD samples in psychology (Henrich et al., 2010), despite the likelihood that cultural orientations shape the relational correlates of mindfulness. These cultural explanations remain speculative, as the present study did not directly assess cultural orientation or related constructs; they are offered as possible directions for future investigation rather than as established explanations for our findings.

Lastly, the choice of measure may be relevant. As noted by several researchers (Kimmes et al., 2025; Quinn-Nilas, 2020), the MAAS (Brown and Ryan, 2003), though widely used, has been criticized for construct validity concerns, reliance on negatively worded items, and its narrow focus on perceived inattention rather than multiple mindfulness facets (Van Dam et al., 2010). In contrast, multifaceted scales such as the Five Facets Mindfulness Questionnaire (FFMQ; Baer et al., 2008) may better capture the construct and show different associations with relationship outcomes (Quinn-Nilas, 2020). However, even the FFMQ has shown weaker or inconsistent links compared to a relationship-specific measure (e.g., McGill et al., 2022). Taken together, these considerations suggest that the link between dispositional mindfulness and relationship satisfaction may vary depending on individual, relational, cultural, and methodological factors.

#### 4.3.2 Self-compassion

In the current study, contrary to our expectations, we found that self-compassion was not significantly associated with relationship satisfaction. Because preliminary analyses revealed gender differences, gender was included as a control variable in the model and it was significantly related to self-compassion, with men reporting higher levels than women, consistent with prior findings (Yarnell et al., 2015).

A considerable body of research has nonetheless demonstrated significant positive associations between self-compassion and romantic relationship functioning. For instance, self-compassionate individuals were perceived by their partners as warmer and more affectionate (Neff and Beretvas, 2013), reported greater authenticity and relational well-being during conflicts (Yarnell and Neff, 2013), and showed more stable marital satisfaction over time (Baker and McNulty, 2011). At the same time, however, findings are not uniform.

Similar to our results, non-significant associations have been reported previously. For example, Maher and Cordova (2019) found no link between self-compassion and relationship satisfaction among meditators. Likewise, Ciarrochi et al. (2024), in a daily diary study, found that self-compassion did not predict how attracted partners felt to one another across days. In addition, Baker and McNulty (2011) observed that among men who lacked motivation to correct interpersonal mistakes, higher self-compassion was linked to poorer marital outcomes. These results suggest that the relational benefits of self-compassion may be context-specific rather than consistent across all outcomes. They also resonate with Bolt et al.’s (2019) argument that while self-compassion may facilitate relationship quality, it does not consistently predict satisfaction, likely because satisfaction is a narrower construct that does not capture nuanced relational aspects. Measures of relationship quality, which assess day-to-day interactional experiences, may be more sensitive to the role of self-compassion than global evaluations of satisfaction. In line with this reasoning, recent studies have shown that self-compassion mediates the link between attachment security and several dimensions of relationship quality, such as emotional intimacy, conflict management, and partner support (Huynh et al., 2022). Taken together, this suggests that self-compassion may relate to healthier relational dynamics and conflict resolution without necessarily translating into higher global satisfaction ratings.

### 4.4 Limitations and future directions

This study drew on a relatively large sample of young adults (*N* = 521), used established measures, and examined attachment, mindfulness, self-compassion, and relationship satisfaction within a single model, offering a comprehensive perspective; nevertheless, the findings should be interpreted in light of several limitations. First, the sampling method was convenience sampling, a non-random approach that may affect the generalizability of the results. The sample also consisted solely of university students, most of whom were women (70%), which further limits generalizability. Future studies could benefit from including more diverse participants across different age groups, relationship types (e.g., dating, cohabiting, married), and cultural contexts to test the theoretical robustness of the proposed model.

Moreover, the study relied on self-report measures, which are susceptible to biases such as social desirability. Additionally, the 5-item short form of the MAAS used in this study, while identified as an efficient and psychometrically strong subset of the full scale (Van Dam et al., 2010) and showing good reliability in the present sample (α = 0.79, ω = 0.80), has not been independently validated in a Turkish sample. Future research should examine its psychometric properties directly in Turkish-speaking populations. Future research may also complement self-reports with behavioral assessments, such as relationship behavior checklists, observational ratings of couple interactions, or partner reports, to obtain a more comprehensive understanding of relationship dynamics. For example, recent research has highlighted the importance of including perceived partner mindfulness alongside self-reports, as mindfulness is often observable through behavior and perceived partner mindfulness has shown stronger associations with marital outcomes than self-reported mindfulness (Kimmes et al., 2025). For example, women’s self-reported relationship mindfulness was unrelated to their own relationship outcomes, whereas their male partners’ reports of the women’s mindfulness levels were predictive. To our knowledge, Kimmes et al.’s (2025) study is the first to examine perceived partner mindfulness in relation to relationship outcomes, underscoring the need for future research to incorporate dyadic designs that measure both self- and partner-reported mindfulness.

Finally, the correlational and cross-sectional design of this study limits our ability to draw conclusions about causal direction. Although we proposed that insecure attachment may be linked to lower relationship satisfaction through reduced mindfulness and self-compassion, it is also plausible that lower satisfaction within a given relationship could be associated with greater reports of attachment insecurity. Indeed, Peters et al. (2024) demonstrated that the implications of attachment insecurity depend not only on individual tendencies but also on partner characteristics, as both one’s own and one’s partner’s insecurity interact to predict relationship outcomes. While attachment theory (Hazan and Shaver, 1987; Mikulincer and Shaver, 2003) and prior longitudinal research (e.g., Davila and Kashy, 2009) support our proposed direction of associations, mediation analyses with cross-sectional data cannot establish temporal precedence. Future studies employing longitudinal or experimental designs are essential to disentangle the temporal ordering of attachment, mindfulness- and compassion-based skills, and relationship well-being.

## 5 Conclusion

This study examined whether dispositional mindfulness, relationship mindfulness, and self-compassion were associated with the well-established link between insecure attachment and romantic relationship satisfaction. Findings indicated that both attachment anxiety and avoidance were associated with lower relationship mindfulness, which in turn was associated with lower relationship satisfaction; however, a significant indirect association was observed only for attachment anxiety, not avoidance, and this association was small in magnitude. These results suggest that relationship mindfulness may be one of several relevant correlates linking attachment anxiety to relationship satisfaction, though the present cross-sectional design does not allow for causal conclusions. Future longitudinal and dyadic research will be essential to clarify the temporal and interpersonal processes underlying these associations.

## Data Availability

The datasets presented in this study can be found in online repositories. The names of the repository/repositories and accession number(s) can be found at: The Open Science Framework: https://osf.io/mc5v2/overview?view_only=1dc927e452f5451b9696b90e71ae9afc.
