# Supplementary material for: Bringing mindfulness to romantic relationships: linking attachment insecurity to young adults’ relationship satisfaction
Source: Front Psychol. 2026 Aug 28;17:1732002. doi: 10.3389/fpsyg.2026.1732002 (PMC13561971; doi:10.3389/fpsyg.2026.1732002)
Supplement: Supplementary file 1 [file Table_1.DOCX]

Supplementary Material

# 1 Supplementary Table

**Supplementary Table 1. Fit Indices for Parceled and Item-Level Measurement Models**

| Models | *χ²* | *df* | *χ²*/*df* | CFI | TLI | RMSEA | SRMR |
| --- | --- | --- | --- | --- | --- | --- | --- |
| Parceled Measurement Model | 856.36 | 309 | 2.77 | .91 | .90 | .06 | .07 |
| Item-level Measurement Model* | 5395.12 | 2000 | 2.70 | .72 | .71 | .06 | .08 |
| **Note**. *N* = 517 and *p* < .001 for both models. Models are estimated using robust maximum likelihood (MLR); robust/scaled fit indices are reported. *χ²* = Chi-Square; df = Degrees of Freedom; CFI = Comparative Fit Index; TLI = Tucker-Lewis Index; RMSEA = Root Mean Square Error of Approximation; SRMR = Standardized Root Mean Square Residual. *Inspection of modification indices for the item-level model indicated two primary sources of local misfit: (1) correlated residuals among items within the same sub-facet of the Self-Compassion Scale (e.g., SC11–SC12, SC3–SC7), consistent with its documented multi-facet structure; and (2) correlated residuals among ECR-R items sharing similar wording or reverse-scoring direction. Given these item-level sources of misfit and the theoretical focus of the present study on the higher-order constructs, we retained the parceled measurement model as primary. | | | | | | | |
